# Renal Amyloid-Associated (AA) Amyloidosis in a Sickle Cell Patient: A Case Report and Literature Review

**DOI:** 10.7759/cureus.36608

**Published:** 2023-03-23

**Authors:** Tsering Dolkar, Henry Mann, Mohammad Salahuddin, Samuel Spitalewitz, Leon Shein

**Affiliations:** 1 Internal Medicine, One Brooklyn Health (OBH) Interfaith Medical Center, Brooklyn, USA; 2 Nephrology, One Brooklyn Health (OBH) Brookdale University Hospital Medical Center, Brooklyn, USA; 3 Nephrology, One Brooklyn Health (OBH) Interfaith Medical Center, Brooklyn, USA

**Keywords:** nephrectomy, gfr, massive proteinuria, sickle cell disease, renal aa amyloidosis

## Abstract

Renal amyloid-associated (AA) amyloidosis is a rare occurrence in sickle cell disease (SCD). Very little literature is available on renal AA amyloidosis in sickle cell disease. Nephrotic range proteinuria is associated with higher mortality among patients with SCD. We present a case of a young reproductive-age African American woman who presented with massive nephrotic range proteinuria. Other more common causes of AA amyloidosis such as immunologic and infectious etiologies were ruled out by history, physical examination, radiologic investigation, and serology. Renal biopsy showed mesangial expansion with Congo red-positive material. Staining for immunoglobulins was negative. Electron microscopy showed nonbranching fibrils. These findings were consistent with AA amyloidosis. This case report adds to the rare findings of renal AA amyloidosis in sickle cell disease. The patient refused any intervention to decrease her Glomerular Filtration Rate (GFR) in the hopes of potentially reversing the disabling proteinuria. We report sickle cell disease presenting with nephrotic syndrome secondary to AA amyloid.

## Introduction

Amyloid-associated (AA) amyloidosis is a deposition of fibrillary fragments of serum amyloid A protein and is associated with chronic inflammatory states that include infectious (HIV), cancers (Lymphoma), and immunologically based illnesses (Lupus, Rheumatoid Arthritis) [1]. Although sickle cell disease is a chronic inflammatory condition, it is rarely associated with AA amyloidosis, with only a handful of cases reported in the literature. We report a case of a patient with homozygous sickle cell disease with multiple recurrent crises presenting with nephrotic range disabling proteinuria whose renal biopsy revealed AA amyloidosis.

## Case presentation

A 42-year-old African-American female with homozygous sickle cell disease presented to Interfaith Medical Center in September 2021 with complaints of generalized body pain and bilateral lower limb swelling. She had been non-compliant to therapy and had multiple painful crises with 20 emergency room visits and five hospitalizations in the past several years. She also had multiple transfusions during these admissions and displayed hemodynamic instability. The patient had a prior history of atraumatic bilateral avascular necrosis of hips in 2015 managed conservatively. There was a prior history of upper GI bleeding which was also managed conservatively. In this admission, the patient denied hip pain, arthralgias, steatorrhea, hematemesis, or hematuria. The patient tested negative for Covid-19. The patient did not have any history of amyloidosis, and the patient was not of Mediterranean descent.

Upon presentation, blood pressure was 90/60 mmHg with a heart rate of 100/min with significant orthostatic hypotension to 80/40 mmHg. The remainder of the physical examination was normal except for severe bilateral lower limb edema and inability to rise to a sitting position without dizziness.

Initial Laboratory findings are shown in Table 1. The patient was transfused for hemoglobin 6.0 g/dL and blood pressure became more acceptable but continued to have orthostatic hypotension.

**Table 1:** Initial laboratory findings.

Investigation	Result	Normal Range
Glucose	116	80-115 mg/dL
Potassium	3.8	4.5-5.5 mmol/L
Sodium	138	135-145 mmol/L
Anion Gap	7	8-16 mmol/L
Blood Urea Nitrogen	11.1	8.4-25.7 mg/dL
Creatinine	1.09	0.72-1.25 mg/dL
Calcium	7.6	8.8-10.0 mg/dL
Albumin	1.9	3.4-5.4 g/dL
Magnesium	1.7	1.7-2.2 mg/dL
Phosphorus	3.2	2.5-4.5 mg/dL
Lactic acid	1.1	0.5-1.9 mg/dL
Creatinine Phosphokinase	18	10-120 mcg/L

History, physical examination, and serologic workup were negative for Systemic Lupus (including ANA, anti-dsDNA, anti-Smith, and anti-SSB antibodies), Rheumatoid arthritis, chronic inflammatory bowel disease, and paraproteinemia. Testing for HIV and hepatitis A, B, and C were negative. C3, C4 were within the normal range of 178 mg/dl (normal 80-200 mg/dl) and 39 mg/dl (12-42 mg/dl), respectively. Thyroid Function Test was normal. Significant laboratory findings during the first admission are shown in Table 2.

**Table 2:** Significant laboratory findings during the first admission.

Since admission	Hemoglobin (Range: 13-17 g/dL)	Hematocrit (Range: 35-46%)	Lactate Dehydrogenase (Range: 140-271 U/L)	Albumin (Range: 3.5-5.5 g/dL)	Total Protein (Range 6.4-8.9 g/dL)
Day 1	6.0	17.5	409	2.7	6.2
Day 3	7.0	21.1	578	1.4	4.0
Day 5	5.3	15.5	302	<0.9	2.5

Urine protein electrophoresis showed significantly elevated levels of total protein, albumin, alpha-1 globulin, alpha-2 globulin, beta globulin, and gamma globulin. Urine analysis showed 4+ proteinuria with urinary protein to creatinine ratio of greater than 18 g of protein per gram of creatinine. Significant proteinuria during first and subsequent admissions is shown in Table 3. The highest being 52 g per day of proteinuria.

**Table 3:** Highest proteinuria during the first, second, and last admissions.

Admission (number)	Urine Protein/Creatinine Ratio (Normal < 0.15 grams/day)
First	18.992
Second	31.479
Last	52.157

**Figure 1:**
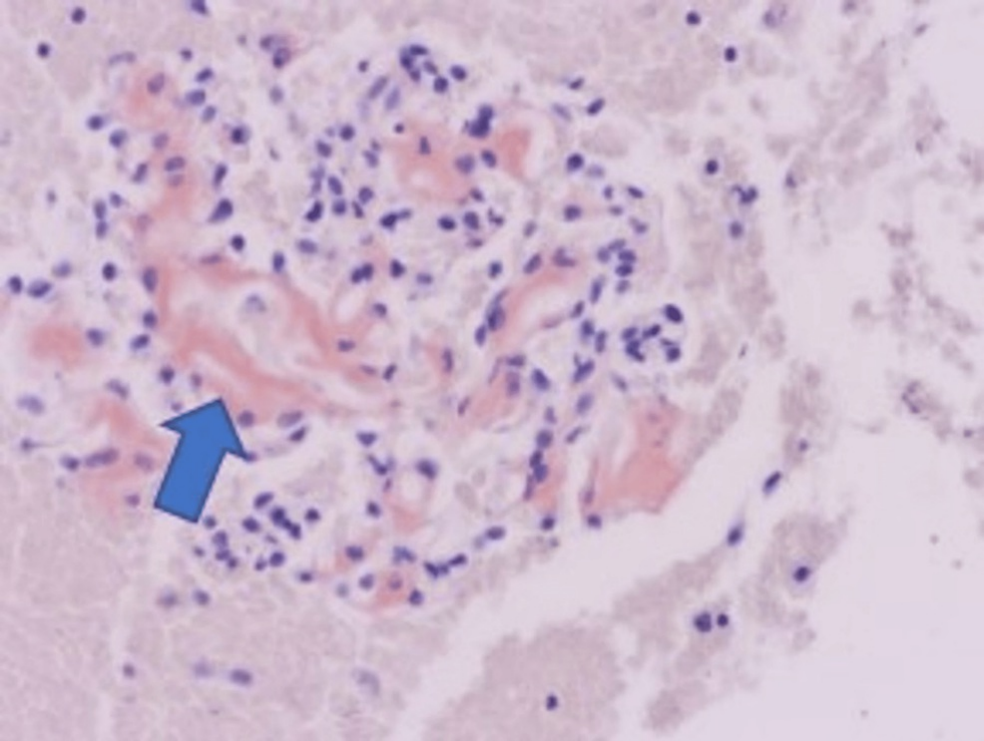
Light microscopy showing mesangial expansion with amorphous eosinophilic material. No glomerular crescents and no necrosis. Mild interstitial fibrosis is present. Congo red positive eosinophilic material (salmon colored) (see arrow) in glomeruli and arterioles with apple green birefringence upon polarization.

**Figure 2:**
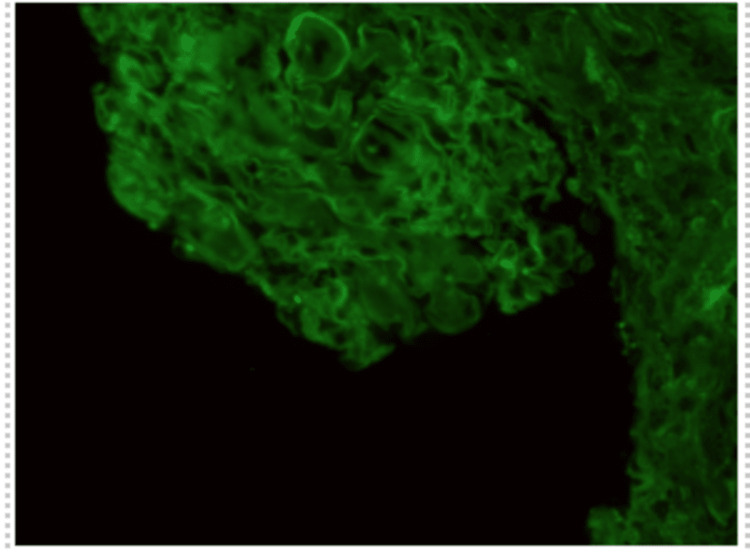
No significant staining for immunoglobulins on immunofluorescence.

**Figure 3:**
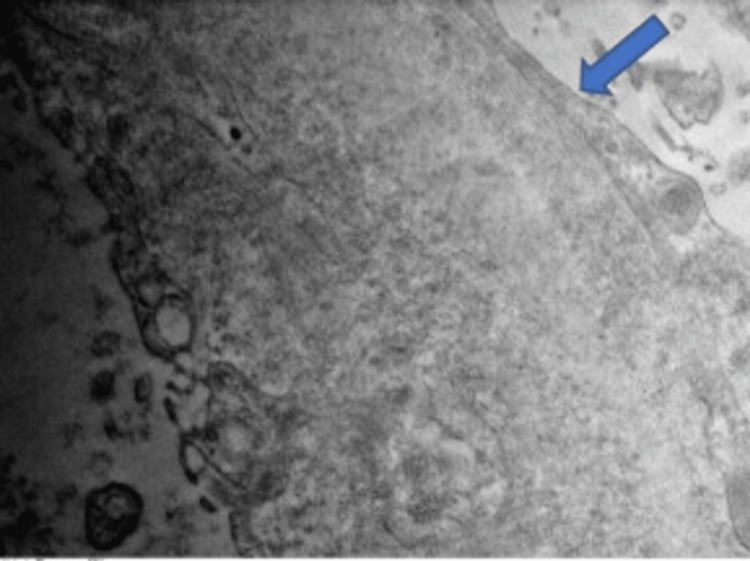
Electron microscopy shows randomly arrayed nonbranching fibrils with deposits in the glomerular membrane. Podocytes show signs of injury with segmental microvillous transformation. There is moderate effacement of foot processes (see arrow). Endothelial cells are normal.

**Figure 4:**
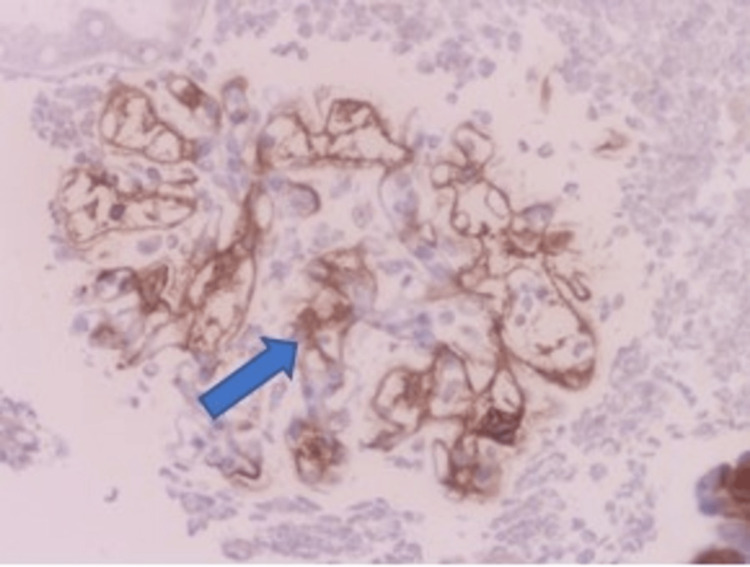
Amyloid A immunostain is positive in the glomeruli.

**Figure 5:**
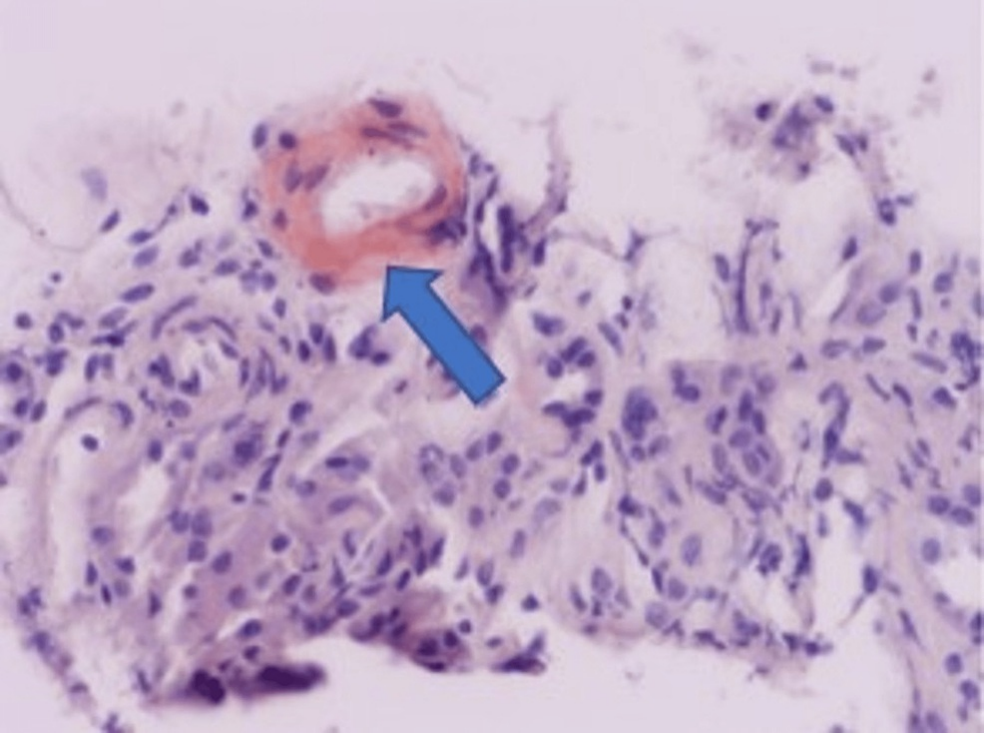
Congo red stain showing salmon-colored areas in the arterioles.

Despite having severe proteinuria, she refused a renal biopsy during the first admission. Radiologic workup included an ultrasound and CT scan of the abdomen and chest that were negative for lymph nodes, tumor, or infections. During the course of second hospitalization, the patient had an episode of sepsis with acute kidney injury associated uremic encephalopathy that required a short course of renal replacement treatment. The patient’s kidney function returned to its baseline spontaneously and renal replacement therapy was discontinued. To delineate the cause of the ongoing nephrotic syndrome, the patient finally consented for a renal biopsy, performed on September 29, 2021. Light microscopic and electron microscopic images are presented below (Figures 1-3). Amyloid A immunostain was shown to be positive in the glomeruli (Figure 4). Congo red staining showed salmon-colored areas in arterioles (Figure 5). Light microscopy revealed mesangial expansion with amorphous material. Congo red was positive. There was no significant staining for immunoglobulins on immunofluorescence. Amyloid A immunostain was positive in the glomeruli. An electron microscopy showed random, nonbranching fibrils. These findings were consistent with AA amyloidosis.

A diagnosis of AA amyloidosis was made. The patient remained severely nephrotic and hemodynamically compromised. Serum creatinine and Glomerular Filtration Rate (GFR) remained stable throughout the course of second admission. Nephrectomy to decrease total protein excretion was considered as an option but the patient adamantly refused. Iatrogenic efforts with angiotensin-converting enzyme (ACE) inhibition, angiotensin receptor blocker (ARB) and glucose transport inhibitors were not considered because of hemodynamic compromise. In the hopes of diminishing her severe life-threatening proteinuria, the patient allowed initiation of a course of cyclosporine, known to stabilize podocytes and cause reversible decline in GFR through vasoconstriction. However, shortly after beginning cyclosporine, the patient signed out against medical advice (AMA), preventing a complete assessment of response to treatment with calcineurin inhibition.

She was subsequently admitted in two other hospitals for similar complaints of sickle cell crises and bilateral leg swelling but also signed AMA. She was readmitted to Interfaith Medical Center for the third time within seven months, in January 2022 for Covid-19 infection at which time she had a fatal cardiac arrest secondary to acute hypoxic respiratory failure.

## Discussion

AA amyloidosis is more often associated with chronic inflammatory conditions such as Crohn’s disease, Rheumatoid Arthritis, Ankylosing Spondylitis, Lupus Nephritis, and Familial Mediterranean Fever (FMF) [1]. Therapy consists of treatment of the underlying disease and, in FMF, colchicine to prevent further formation. Sickle cell crises represent chronic inflammation and oxidative stress [2]. Sickle cell anemia is associated with various renal lesions and has thus been termed sickle cell nephropathy [3]. The involvement includes papillary necrosis, interstitial nephritis, associated renal insufficiency, nephrogenic diabetes insipidus, and glomerular lesions [3]. Focal Glomerulosclerosis (including rarely the collapsing variety) and Membranoproliferative Glomerulonephritis have been reported as the most common glomerular diseases causing nephrotic syndrome in sickle cell anemia [4]. In most cases, however, this is thought to be a secondary variety of focal glomerulosclerosis rather than primary with glomerular hyperfiltration and hypertrophy. Amyloidosis is only rarely reported.

Even though sickle cell anemia is associated with chronic inflammation in patients with multiple sickle crises, amyloidosis has been rarely reported in sickle cell disease [5,6]. The kidney is the major involved organ, with proteinuria as the first clinical manifestation, warranting a renal biopsy [5]. Clinically significant renal involvement occurs more frequently in sickle cell disease than in sickle cell trait or combined hemoglobinopathies [7]. However, profound proteinuria over 20 grams is virtually non-existent in sickle cell patients and would therefore strongly suggest an additional renal pathology [8].

Our patient had a history of multiple sickle cell crises with frequent hospitalizations in the recent past prior to presentation at our institution. AA amyloidosis was demonstrated by renal biopsy with positive Congo red staining and apple green birefringence with amyloid A staining on immunofluorescence and compatible electron microscopic findings. Other causes of AA amyloidosis were ruled out by history, clinical and laboratory data. We postulate that because of the multiple hospitalizations for sickle cell crisis and severe ongoing inflammation, our patient was prone to develop AA amyloidosis. The patient had a history of avascular necrosis but no active inflammation or local pain was demonstrated. In addition, workups for other chronic inflammatory state such as Crohn's disease and Rheumatoid Arthritis were negative. Vasculitis, hepatitis, lupus nephritis, HIV nephropathy, and drug-induced nephropathy were also ruled out. Therefore, by exclusion, it appears that AA amyloidosis was associated with the patient's antecedent diagnosis of sickle cell disease.

The patient had severe hypoalbuminemia with disabling edema. Moreover, the patient had a prior history of GI bleeding, chronic persistent hemodynamic instability, and relative hypotension that prevented the institution of nonsteroidal anti-inflammatory drugs, ACE inhibition or ARB therapy that may have mitigated the proteinuria, while refusing a more permanent reduction in GFR by nephrectomy or embolectomy. The patient had enough preserved kidney function such that renal replacement therapy was not necessary. The patient ultimately allowed initiation of cyclosporine, known to diminish proteinuria by causing vasoconstriction and a reversible decrease in GFR resulting in stabilization of podocytes, however, she signed out AMA shortly after its institution. The treatment of AA amyloidosis is not well studied, unlike light-chain renal amyloidosis, which has shown encouraging results with chemotherapy [9].

## Conclusions

To our knowledge, this is the seventh reported case of AA amyloidosis associated with sickle cell disease. Therefore, we believe that Sickle Cell Disease needs to be considered in the differential diagnosis of nephrotic syndrome in sickle cell patients. Other than diminishing the number of sickle cell crises that might prevent further AA amyloid deposition and progressive renal dysfunction, efforts to decrease proteinuria should also be undertaken in order to reduce the associated disabling edema, potential hemodynamic instability, and life-threatening infections. The approach may include ACE inhibition, ARB use, glucose transport inhibitors, nonsteroidal anti-inflammatory drugs and calcineurin inhibitors, and lastly, possible nephrectomy if all else fails. It is unclear what should be the best treatment strategy for renal AA amyloidosis in a sickle cell patient. Further investigation is necessary but difficult when there are so few reported cases.
